# Biomechanical Analysis of Rotator Cuff Repair: Comparison of Sutures and Fixation Techniques in a Synthetic Model

**DOI:** 10.7759/cureus.116784

**Published:** 2026-09-23

**Authors:** Murilo Baracat Cortese Conde, Felipe Mendes Fernandes, Sergio Keiiti Ozima Filho, Rodrigo Augusto C Ferreira, Henrique Cristovao Ferreira, Vitor Elias Correa

**Affiliations:** 1 Orthopaedics and Traumatology, Fundação Padre Albino, Catanduva, BRA; 2 Medicine, Faculdade de Medicina de Jundiaí, Jundiaí, BRA; 3 Orthopaedic Surgery, Fundação Padre Albino, Catanduva, BRA

**Keywords:** biomechanics, irreparable, rotator cuff, sutures, tensile strength

## Abstract

Introduction

Irreparable rotator cuff tears represent one of the greatest challenges in contemporary orthopedic surgery, imposing significant functional limitation and requiring reconstruction strategies with high mechanical fixation strength. The choice between different suture materials and anchoring techniques directly influences the initial biomechanical stability of the repair, determining construct safety during the critical period of biological healing.

Objective

To evaluate and compare the load-to-failure of four tendon repair configurations in humeral head synthetic models (Sawbones®; Pacific Research Laboratories, Inc., Vashon, WA, USA), combining two surgical sutures - Ethibond® USP 5 (Ethicon Inc., Raritan, NJ, USA; braided polyester) and FiberWire® USP 2 (Arthrex Inc., Naples, FL, USA; ultra-high-molecular-weight polyethylene) - with two fixation techniques: double metal anchor and transosseous tunnel.

Methods

Linear tensile mechanical tests to failure were performed on 20 shoulder models, with five samples per experimental group. The tendon was simulated using synthetic leather strips (corino), secured with a five-pass epitendinous running suture. Maximum load to failure was measured with a calibrated dynamometer in kilogram-force (kgf) and converted to Newtons (N). Inclusion criteria restricted the analysis to failures exclusive to the suture material, excluding any structural construct failure.

Results

All configurations exceeded the 200 N biomechanical threshold considered safe for early rehabilitation. The Ethibond® transosseous tunnel group showed the highest mean strength (357.55 N; SD ± 46.7), followed by FiberWire® with double anchor (330.88 N; SD ± 18.3), transosseous FiberWire® (296.36 N; SD ± 8.6), and Ethibond® with double anchor (293.41 N; SD ± 43.9). Between-group Welch's t-tests showed a trend toward higher strength for the Ethibond® transosseous configuration versus Ethibond® double anchor (p = 0.056), and a significant difference between FiberWire® double anchor and FiberWire® transosseous (p = 0.010); pooled main effects of suture material and fixation technique alone were not significant, suggesting an interaction between suture caliber and fixation geometry.

Conclusion

In this synthetic model, the transosseous technique combined with high-caliber polyester suture produced the greatest mean mechanical strength, although the advantage over anchor fixation did not reach statistical significance. Surgeons may achieve biomechanically safe and cost-effective fixation using transosseous tunnels with Ethibond® USP 5, reserving anchors and FiberWire® for cases in which tear geometry precludes tunnel creation.

## Introduction

The biomechanics of rotator cuff repair remains one of the most dynamic and challenging fields of study within orthopedic and trauma surgery. Injuries affecting this tendinous complex of the shoulder have a significant impact on joint function and patients' quality of life [1]. The success of surgical treatment, whose primary goal is to restore shoulder anatomy and function, critically depends on the initial mechanical stability of the surgical construct [2]. This stability must be robust enough to withstand the tensile forces generated by early rehabilitation, ensuring that the tendon remains firmly fixed to the bone during the period required for biological tissue healing [2,3].

In the setting of rotator cuff fixation, the interface between bone, tendon, and suture material constitutes a complex system subject to failure at multiple points [3]. Historically, non-absorbable braided polyester sutures, commercially represented by Ethibond® (Ethicon Inc., Raritan, NJ, USA), became established as the standard in orthopedic practice due to their pliability and knot security [4]. However, advances in materials engineering introduced ultra-high-molecular-weight polyethylene (UHMWPE) sutures, such as FiberWire® (Arthrex Inc., Naples, FL, USA), which exhibit substantially greater tensile strength and lower deformation under load [4,5]. Transitioning between, or choosing among, these materials requires a thorough understanding of their biomechanical properties under stress.

Beyond the nature of the suture itself, the surgical technique used for bone fixation is a vital determinant of repair longevity. The traditional transosseous tunnel technique offers a favorable biological distribution of forces but demands greater technical skill [6]. In contrast, the development of suture anchors revolutionized shoulder surgery, simplifying the procedure and concentrating fixation points, although this introduces new stress vectors and potential failure modes, such as anchor pull-out or suture cutting through the device's eyelet [6,7].

Experimental models and biomechanical simulators play a fundamental role in elucidating these variables, allowing isolation of mechanical factors without the uncontrollable biological variables present in vivo [8]. The use of synthetic polyurethane bone blocks (Sawbones®; Pacific Research Laboratories, Inc., Vashon, WA, USA) standardizes the density and mineral strength of the greater humeral tuberosity, mitigating the bias introduced by the heterogeneous bone quality found in cadaveric specimens [8,9]. In parallel, the use of synthetic substrates or tissues of controlled density, such as synthetic leather (corino), serves as a practical and reproducible substitute for simulating the thickness and tensile strength of rotator cuff tendon tissue.

Within this context, the present research aims to quantitatively evaluate and compare the mechanical tensile strength of different repair configurations. Through a controlled experimental model, the study analyzes the performance of Ethibond® and FiberWire® sutures arranged in two distinct fixation techniques: suture anchors and bone tunnels. By comparing the cross-combinations of these materials and methods, the research seeks to provide objective biomechanical evidence to help orthopedic surgeons choose the safest and most effective strategy for rotator cuff reconstruction.

Rationale

Irreparable rotator cuff tears represent one of the greatest challenges in shoulder surgery, predominantly affecting elderly patients and imposing significant functional limitation. The absence of viable tendon tissue for direct primary repair requires surgeons to master alternative reconstruction techniques, in which the choice of fixation method plays a determining role in the biomechanical - and consequently clinical - outcome.

Among the fixation methods most commonly used in orthopedic practice, anchor fixation and transosseous fixation stand out. Although both techniques are supported in the literature, controversy persists regarding their relative biomechanical superiority, particularly in scenarios of compromised bone quality, which are frequent among patients with chronic, irreparable tears. The quality of anchor fixation depends on multiple factors, including the tensile strength of the suture and the interaction between the anchor and the suture material, both of which are determinants of repair success [10]. A direct, controlled comparison between these methods is therefore essential to support surgical decision-making with a higher level of evidence.

In addition to the fixation technique, the choice of suture material directly influences tensile strength, cyclic fatigue behavior, and construct safety [3]. Ethibond®, made of braided polyester, and FiberWire®, composed of high-molecular-weight polyethylene with a polyester core, have distinct mechanical properties that may significantly impact fixation integrity. Although both are widely used, direct comparative studies between these materials in the context of irreparable rotator cuff tears remain scarce in the literature [11,12]. Furthermore, current concepts in arthroscopic rotator cuff repair emphasize that understanding material fatigue and structural configuration is paramount to avoiding construct failure [13].

Objectives

General Objective

To biomechanically evaluate the strength of fixation methods using conventional metal anchors and transosseous tunnels with Ethibond® and FiberWire® sutures in tendon repairs performed on Sawbones® humeral head models.

Specific Objectives

To compare the mechanical strength between conventional metal anchor and transosseous tunnel fixation methods; to evaluate the difference in strength between Ethibond® and FiberWire® sutures; to analyze the biomechanical behavior of epitendinous running sutures subjected to tension; to identify the predominant mode of failure in each fixation technique; and to determine which suture-fixation method combination provides the greatest biomechanical stability in tendon repair.

## Materials and methods

Study design

This study was designed as a controlled, comparative, in vitro biomechanical investigation using a synthetic bench-top model of rotator cuff repair. A 2×2 factorial arrangement was used, cross-combining two suture materials (Ethibond® USP 5 and FiberWire® USP 2) with two fixation techniques (double metal anchor and transosseous tunnel), producing four experimental groups of five specimens each (n = 20 total, five samples per group). All specimens underwent linear (uniaxial) tensile loading to failure. Data collection took place from March to May 2026.

Study model and specimen preparation

Because this research used exclusively synthetic surrogate materials rather than human or animal tissue, the unit of analysis was the individual construct (specimen) rather than a living participant; this subsection therefore describes the experimental model in place of a conventional “Study Participants” subsection.

The models were shoulder simulators (Sawbones®) fitted with synthetic leather strips simulating the rotator cuff tendons. To ensure test fidelity, a support structure made of pine wood and fixed pulleys was built to redirect the applied traction to a steel cable connected to the synthetic leather, simulating the torn supraspinatus tendon.

The cable was subjected to constant manual traction using a dynamometer that registered, in kgf, the force required to rupture the tested suture. The repair configuration consisted of fixing the tested suture to an anchor or bone tunnel, followed by five passes of a running suture through the leather, and fixation to a subsequent anchor or bone tunnel positioned 3 cm sagittally from the first.

The sutures tested were Ethibond® USP 5 and FiberWire® USP 2. The anchor tested was a 5.0 mm metal anchor. Bone tunnels were created with a 3 cm sagittal distance between them and a 1.5 cm coronal distance within each bone tunnel.

The dynamometer used was a hand-grip strength testing device, graduated in kgf with 0.1 kgf increments and a margin of error of ±0.5 kgf, adapted with steel cables attached via a shackle so that the force exerted at the handle was identical to that exerted on the synthetic leather.

The inclusion criteria were all tests in which failure occurred exclusively at the suture material.

The exclusion criteria were any failure originating from other structures of the construct, such as untied knots, ruptured synthetic leather, anchor pull-out, bone tunnel failure, scapular fracture, or overall construct failure.

Ethical considerations

This study did not involve human participants, human tissue, animal subjects, or animal tissue; all testing was performed exclusively on synthetic bone (Sawbones®) and synthetic tendon surrogate (corino leather) models. Accordingly, institutional review board and ethics committee approval, as well as informed consent, were not required, consistent with the authors' disclosures in the Additional Information section.

Statistical analysis

Descriptive statistics (mean and standard deviation) were calculated for the maximum load to failure (in Newtons) in each of the four experimental groups (n = 5 per group). Raw values, collected in kilogram-force (kgf), were converted to Newtons using the standard conversion factor (1 kgf ≈ 9.81 N).

Between-group comparisons were performed with the unpaired, two-tailed Welch's t-test, which does not assume equal variances and is appropriate given the heterogeneous standard deviations observed among groups. Pairwise comparisons were performed between suture materials within each fixation technique and between fixation techniques within each suture material. Pooled main-effect comparisons (suture material irrespective of technique, and fixation technique irrespective of suture material) were performed by combining the relevant groups (n = 10 each) using the same approach. A p-value < 0.05 was considered statistically significant.

## Results

Linear tensile mechanical tests to failure were performed on 20 humeral head simulators (Sawbones®) combined with synthetic leather tendons, following the epitendinous running suture protocol with five passes [1]. Raw data, collected in kilogram-force (kgf), were converted to Newtons (N) using the standard conversion factor (1 kgf ≈ 9.81 N) to allow comparison with the physiological loading parameters described in the literature [3,10].

All 20 tested specimens met the pre-specified inclusion criteria: failure occurred exclusively at the suture material (complete suture rupture) in every case. No anchor pull-out, bone tunnel failure, knot slippage, or rupture of the synthetic leather tendon substitute was observed in any group, confirming that the failure mode analyzed throughout the study was suture-specific across all four configurations, in keeping with the specific objective of identifying the predominant mode of failure.

Strength by group and material

Quantitative analysis showed that all tested configurations exceeded the 200 N biomechanical threshold, identified as the minimum load required to withstand the stresses of activities of daily living during the initial healing phase [1,6]. Descriptive results for all four groups are summarized in Table 1.

**Table 1:** Mean maximum load to failure (N) by experimental group SD = Standard Deviation; N = Newtons; n = number of samples

Test Configuration	Samples (n)	Mean Load (N)	Standard Deviation (N)
Ethibond® Double Anchor	5	293.41	± 43.9
FiberWire® Double Anchor	5	330.88	± 18.3
Ethibond® Transosseous	5	357.55	± 46.7
FiberWire® Transosseous	5	296.36	± 8.6

Transosseous (Intraosseous) Tunnel Group

The repair using the transosseous tunnel technique with Ethibond® USP 5 showed the highest mean strength in the study, reaching 357.55 N (SD ± 46.7). In contrast, the same technique with FiberWire® USP 2 achieved a mean of 296.36 N (SD ± 8.6). The larger caliber of Ethibond® (USP 5) in this configuration was observed to compensate for the lower intrinsic strength of polyester compared with the polyethylene blend [5,10].

Double Anchor Group

In this technique, FiberWire® USP 2 demonstrated superiority over Ethibond®, with a mean maximum load of 330.88 N (SD ± 18.3), compared with 293.41 N (SD ± 43.9) for the Ethibond® group. FiberWire® values approached the upper limit reported for this material alone in pure tensile testing [3,5].

Statistical comparisons

**Figure 1:**
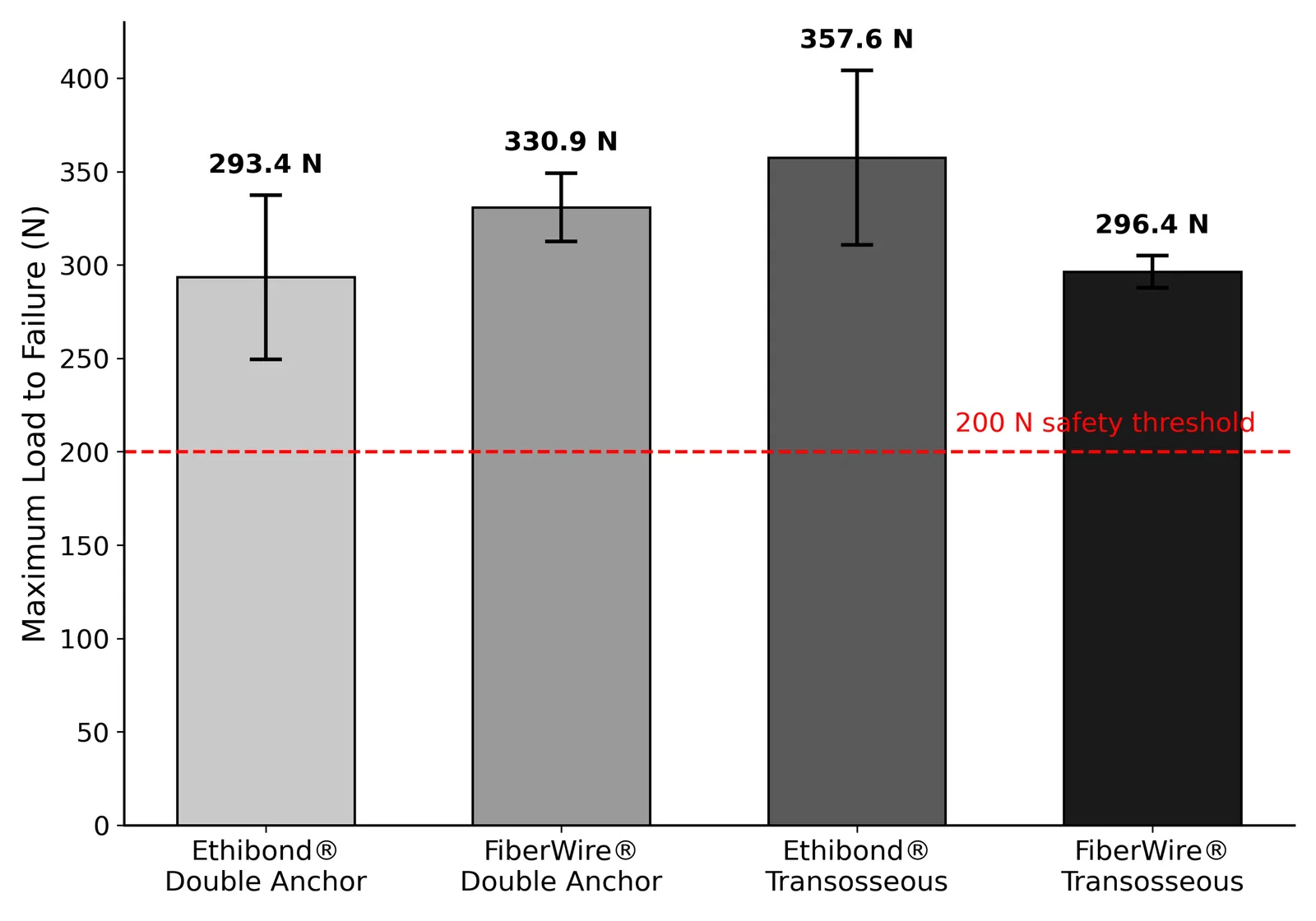
Mean maximum load to failure (N) by experimental group, with error bars representing ± 1 standard deviation The dashed red line indicates the 200 N threshold considered the minimum load required to withstand the stresses of activities of daily living during the initial healing phase.

Between-group comparisons using Welch's two-tailed t-test are summarized in Table 2 and Figure 1. The overall highest-strength configuration, Ethibond® transosseous, showed a trend toward greater strength than Ethibond® double anchor, corresponding to a 21.9% relative increase in mean load, but this difference did not reach conventional statistical significance (t = 2.24, p = 0.056), likely reflecting the limited statistical power of the small per-group sample size (n = 5). Within the transosseous group, Ethibond® was significantly stronger than FiberWire® (t = 2.88, p = 0.042). Within the double-anchor group, FiberWire® showed a numerically higher mean than Ethibond®, but the difference was not statistically significant (t = 1.76, p = 0.135). FiberWire® double anchor was significantly stronger than FiberWire® transosseous (t = 3.82, p = 0.010), indicating that, for this UHMWPE suture, fixation technique had a measurable effect on performance.

**Table 2:** Between-group statistical comparisons (Welch's two-tailed t-test) *Calculated from the group mean, standard deviation, and sample size (n = 5/group) reported in Table 1. p < 0.05 is considered statistically significant.

Comparison	t	df	p-value*
Ethibond® Transosseous vs. Ethibond® Double Anchor	2.24	8.0	0.056
FiberWire® Double Anchor vs. Ethibond® Double Anchor	1.76	5.3	0.135
Ethibond® Transosseous vs. FiberWire® Transosseous	2.88	4.3	0.042
FiberWire® Double Anchor vs. FiberWire® Transosseous	3.82	5.7	0.010
Suture material, pooled (Ethibond® vs. FiberWire®)	0.64	12.0	0.537
Fixation technique, pooled (Anchor vs. Transosseous)	0.80	17.4	0.435

When suture material was pooled across fixation technique, no significant overall difference was found between Ethibond® (pooled mean 325.5 N) and FiberWire® (pooled mean 313.6 N; p = 0.537). Likewise, when fixation technique was pooled across suture material, no significant overall difference was found between double anchor (pooled mean 312.1 N) and transosseous fixation (pooled mean 327.0 N; p = 0.435). Taken together, these findings indicate that neither suture material nor fixation technique alone was an independent determinant of construct strength; rather, the data suggest an interaction effect, in which the combination of high-caliber polyester suture with transosseous fixation and of UHMWPE suture with anchor fixation each optimizes performance within their respective configuration.

## Discussion

The results demonstrate that fixation geometry and suture caliber are critical determinants of the final strength of the repair. The most notable finding was the trend toward superiority of the transosseous tunnel technique with Ethibond® USP 5, which outperformed the double anchor technique by approximately 21.9% in mean load to failure, although this difference approached but did not reach statistical significance (p = 0.056). This finding is broadly consistent with the literature indicating that transosseous tunnels allow a more favorable distribution of forces at the bone-tendon interface [6,9].

Although FiberWire® has a UHMWPE composition with intrinsically greater strength [5], the use of a significantly larger-caliber polyester suture (USP 5 versus USP 2) neutralized this technological advantage in the transosseous model. In addition, the lower abrasiveness of polyester may have reduced the progressive cutting effect on the synthetic leather (cheese-wiring), allowing the system to reach higher loads before failure [3]. In the double anchor group, the stiffness of FiberWire® adapted better to the focal fixation points, resulting in a numerically greater mean load compared with Ethibond® in the same configuration, although this difference did not reach statistical significance (p = 0.135) [1,6].

Comparison with the existing literature

The present findings are consistent with, and extend, previous comparative work on suture and fixation biomechanics. Guimarães and Carvalho Júnior [1] tested Ethibond® #2 and FiberWire® #2 with titanium suture anchors and reported considerably lower maximum loads (around 230-298 N) than those observed here for either suture at USP 5/USP 2 caliber, and found that Ethibond® #2 failed at loads below those considered necessary for safe rotator cuff fixation; this contrast underscores how suture caliber, rather than material alone, can be decisive for construct strength, matching the compensatory effect of high-caliber Ethibond® USP 5 identified in the present study. Similarly, Gomide et al. [3], testing isolated suture specimens in pure tension, found FiberWire® USP 2 to be the strongest suture evaluated (mean failure load of approximately 240 N), a value considerably lower than the loads reached by the same suture once incorporated into either fixation construct in the present study; this suggests that the surrounding fixation geometry (anchor or bone tunnel) and the multi-pass running suture configuration meaningfully amplify the effective strength of the repair beyond the isolated tensile capacity of the suture material itself.

Wüst et al. [4] similarly demonstrated the superior isolated mechanical properties of UHMWPE polyblend sutures such as FiberWire® over braided polyester, which aligns with the numerically higher performance of FiberWire® observed here in the anchor configuration, but does not, by itself, predict superiority once caliber and fixation geometry are also varied, as shown by the present transosseous results. In the clinical setting, Na et al. [6], in a meta-analysis comparing arthroscopic transosseous anchorless repair with suture anchor repair for rotator cuff tears, found comparable clinical and structural outcomes between the two techniques; the lack of a significant overall difference between fixation techniques when pooled across suture material in the present biomechanical model (p = 0.435) mirrors this clinical equivalence and lends biomechanical support to the pragmatic, cost-driven selection of transosseous fixation suggested by those clinical results. Gerber et al. [7], in a now-classic study using osteoporotic cadaveric specimens, reported considerably lower failure loads for both transosseous sutures and anchor fixation (approximately 140 N) than found in the present synthetic model; this discrepancy highlights the importance of bone mineral density as a variable that standardized synthetic bone blocks such as Sawbones® deliberately eliminate, and suggests that the safety margins demonstrated here may not be fully generalizable to patients with compromised bone quality.

More recently, Haroun et al. [12], in a biomechanical animal study comparing four suture configurations for rotator cuff repair, and Lee et al. [13], in a review of current concepts in arthroscopic rotator cuff repair, both emphasized that construct performance depends on the interaction between suture material, suture configuration, and fixation method rather than on any single variable in isolation - a conclusion directly supported by the present finding that neither suture material nor fixation technique alone produced a statistically significant main effect, while specific material-technique combinations did differ meaningfully. Meyer et al. [8] additionally described suture erosion through the anchor eyelet as a clinically relevant failure mode specific to anchor-based repairs; because the present inclusion criteria restricted analysis to suture-material failure and excluded eyelet-related or anchor pull-out failures, the comparative advantage of FiberWire® in the anchor group observed here should be interpreted as an estimate of intrinsic suture performance rather than of overall clinical anchor-construct durability, which would also depend on eyelet-related wear mechanisms not captured by this experimental design.

This study has several limitations that should be considered when interpreting these results. First, the synthetic bench-top model, while advantageous for standardizing bone density and eliminating inter-specimen biological variability, cannot fully reproduce the viscoelastic behavior, vascularity, and biological healing response of native tendon and bone, and the corino leather substitute, selected as a practical, low-cost, and reproducible material for standardizing suture-holding tissue across specimens, differs structurally from human rotator cuff tissue. Second, testing consisted of a single ramped pull to failure rather than cyclic sub-maximal loading, which more closely reflects the repetitive loading pattern experienced by a repair construct during early postoperative rehabilitation and may reveal different failure behavior than a single supramaximal load. Third, the two suture materials were compared at different calibers (Ethibond® USP 5 versus FiberWire® USP 2), which reflects common surgical practice but confounds the independent contributions of polymer composition and suture diameter to the observed differences. Fourth, the small sample size (five specimens per group) limited the statistical power of the comparisons, as reflected in the borderline significance of the main finding (p = 0.056) and the wide confidence intervals implied by the standard deviations in Table 1; a larger sample would be needed to confirm whether this trend represents a true biomechanical difference. Fifth, force was applied manually using a hand-grip dynamometer rather than a servo-controlled materials testing machine, which may have introduced variability in loading rate between specimens despite the fixed pulley system used to standardize the direction of traction. Finally, only a single anchor size and a single tunnel geometry were tested, and the results may not generalize to other implant designs, tunnel configurations, or suture-passing techniques used in clinical practice.

## Conclusions

In this synthetic biomechanical model, all four suture-fixation configurations exceeded the 200 N threshold considered safe for early rehabilitation. The transosseous tunnel technique combined with high-caliber Ethibond® USP 5 produced the greatest mean load to failure, although this advantage over double-anchor fixation approached but did not reach statistical significance (p = 0.056). FiberWire® USP 2 performed more consistently, and significantly better, with anchor fixation than with transosseous tunnels. No independent main effect of suture material or fixation technique alone was found, indicating that construct strength depends on the specific pairing of suture caliber and fixation geometry rather than on either factor in isolation. These findings support transosseous tunnel fixation with high-caliber polyester suture as a biomechanically sound and cost-effective option, reserving metal anchors and technologically advanced sutures for tears whose geometry precludes tunnel creation; clinical and cadaveric validation of these synthetic-model findings is warranted.
